# Iatrogenic central retinal artery occlusion after carotid body tumor embolization and excision

**DOI:** 10.3205/oc000060

**Published:** 2017-03-24

**Authors:** Carlos M. Rangel, Sergio Jaramillo, Clara L. Varón, Angélica M. Prada

**Affiliations:** 1 Retina and Vitreous Department, Fundación Oftalmológica de Santander Clínica Carlos Ardila Lulle, Bucaramanga, Colombia; 2 Retina and Vitreous Department, Clínica Oftalmológica del Café, Manizales, Colombia; 3 Fundación Oftalmológica de Santander Clínica Carlos Ardila Lulle, Bucaramanga, Colombia

**Keywords:** carotid body tumor, central retinal artery occlusion, embolization, Iatrogenic, paraganglioma, polyvynil alcohol, trys-acryl gelatin

## Abstract

**Objective:** To report a case of iatrogenic central retinal artery occlusion after embolization and surgical resection of carotid body paraganglioma.

**Methods:** Case report

**Results:** One adult female patient presented with persistent unilateral visual loss after embolization with Embosphere^®^ and Contour^®^ microparticles of carotid body tumor. Fluorescein angiography revealed intraluminal microspheres in the central retinal artery ramifications. OCT revealed intraretinal spherical, hyporeflective particles with posterior shadowing.

**Conclusions:** Central retinal artery occlusion should be assessed as a possible complication after surgical repair of head and neck paragangliomas.

## Introduction

The carotid body is a small ovoid or irregular mass bilaterally situated on the bifurcation of the common carotid artery. It functions as a chemoreceptor to changes in arterial pO_2_, CO_2_, and pH, which induces reflex changes in vasomotor activity and respiration [1]. Carotid body tumors (paraganglioma) are uncommon neoplasms that arise from the paraganglion cells of the carotid body with an incidence of about 1 in 30,000. Although the most common form of head and neck vascular tumor, most vascular surgeons will encounter only a few paraganglioma cases during their career [2]. They are highly vascularized tumors with feeder vessels mainly originating from the ascending pharyngeal artery, the occipital branch, posterior auricular branch, superior thyroidal branch and lingual branch of the external carotid artery (ECA). Other branches originated from the internal carotid artery (ICA), the ECA, or the posterior circulation may also supply the tumor as it grows [3]. The mortality rate associated with this tumor is 8% in untreated cases [4]. Paraganglioma may manifest as a cervical mass usually located under the angle of the mandible. Additional symptoms are dizziness, syncope, cranial nerve palsies, hoarseness, sore throat, and dysphagia. Metastasis have also been reported, approximately 6% [5], mainly to the regional lymph nodes [6]. Duplex ultrasound scanning is considered the primary diagnostic test [2]. Angiography, computed tomography (CT) scan, CT angiography, magnetic resonance imaging (MRI) and magnetic resonance angiography are also used for preoperative assessment. Surgical resection remains the mainstay of treatment [2]. However, complications related to the procedure have been described as high as 33% [2]. Cranial nerve damage, stroke, wound haematoma, TIA (transient ischemic attack), horner syndrome, have been described. Additional procedures such as preoperative embolization have been proposed to reduce the incidence of massive intraoperative bleeding and, hence, lower the mortality rate [7]. We describe a patient who suffered permanent loss of vision in one eye after intraarterial embolization with trisacryl gelatin microspheres (Embosphere^®^) and polyvinyl alcohol (PVA), Contour^®^ microparticles (Boston Scientific, Boston, MA) and surgical resection of carotid body paraganglioma.

## Case description

**Figure 1:**
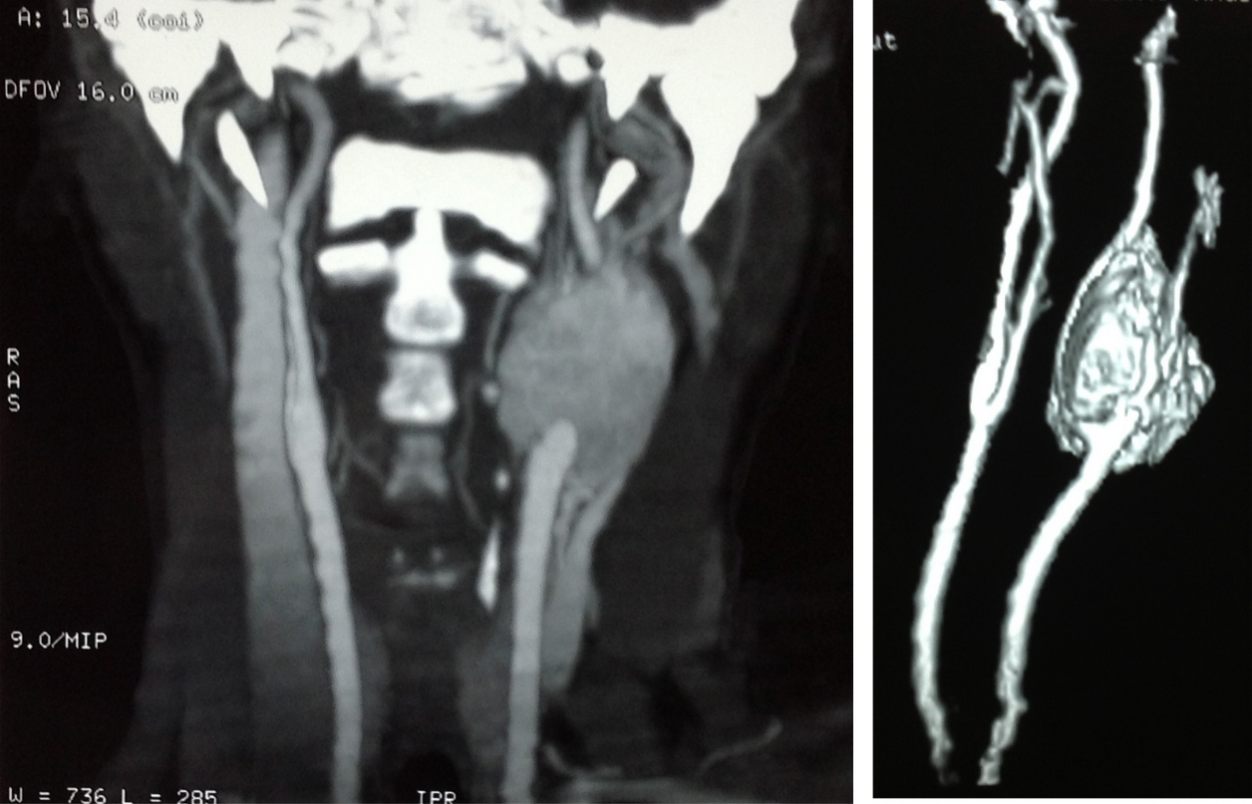
CT angiography reveals a mass on the biphurcation of the left common carotid artery.

A 45-year-old female presented with a history of syncope, headache, and dyspnea for three years. A pulsatile mass was discovered on her left side of the neck. CT angiography revealed a mass located on the left carotid bifurcation classified as Shamblin II (Figure 1 (Fig. 1)). Preoperative embolization was performed the day before surgical resection. Under general anesthesia, transfemoral access was obtained with selective catheterization of internal and external carotid artery. A 5x4 cm tumor was localized at the biphurcation of the left common carotid artery with feeding vessels from the ascending pharyngeal, occipital and superior thyroid arteries. Carotid occlusion test was performed to verify adequate permeability of the circle of Willis. A 6 Fr catheter was advanced into the left common carotid artery with subsequent microcatheterization of the ascending pharyngeal artery. This was followed by embolization with Contour^®^ Polyvynil alcohol embolization particles (Boston Scientific, Boston, MA) of 150–250 microns, and then by injection of Embospheres^®^ (Merit Medical Systems, USA) of 300–500 microns until vessel occlusion was achieved. The same procedure was performed for the occipital artery. The superior thyroid artery could not be embolized because of severe vessel tortuosity. A post-embolization angiography revealed adequate permeability of intracranial and extracraneal vessels. An average decrease of 80% of the tumor’s blood flow was documented. After the procedure was completed, the patient woke up without neurological or visual deficits. The surgical resection was then performed under general anesthesia. A transverse cervical incision and dissection to complete exposure of the tumor was done. The surrounding vascular structures (common carotid artery, internal and external carotid) with extension to the skull base were identified. The hypoglossal nerve, the hypoglossal loop and vagus nerve, were released without severing. The tumor resection was performed with the subadventitial technique [8]. Minimum bleeding occurred and no neurovascular structures were severed. 

**Figure 2:**
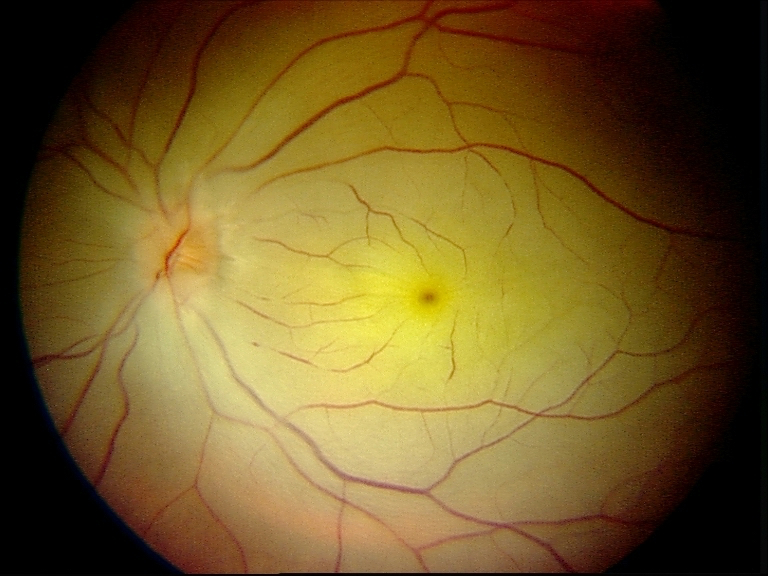
Color photography shows a pale retina with pronounced disturbance of the retinal circulation in the posterior pole.

**Figure 3:**
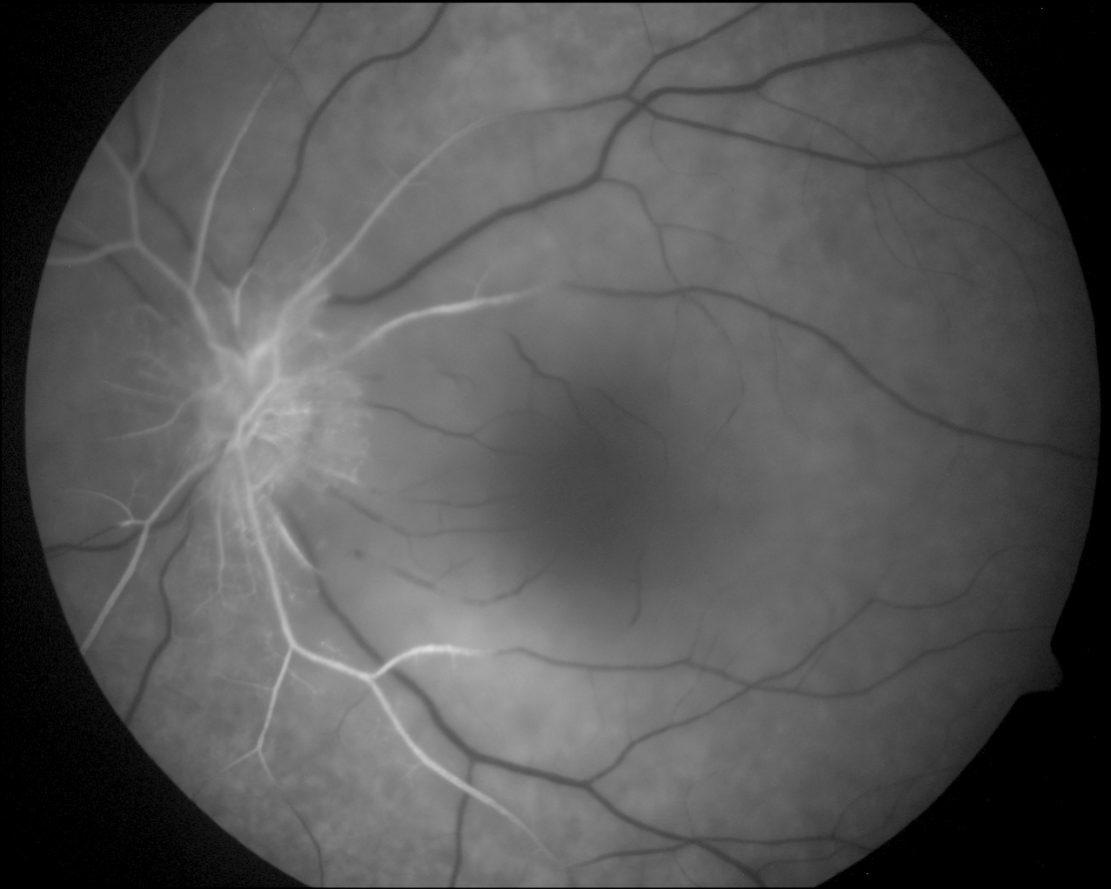
Fluorescein angiography showing abrupt interruption of flow on the ramifications of the central retinal artery and macular ischemia due to short posterior ciliary interruption of flow

**Figure 4:**
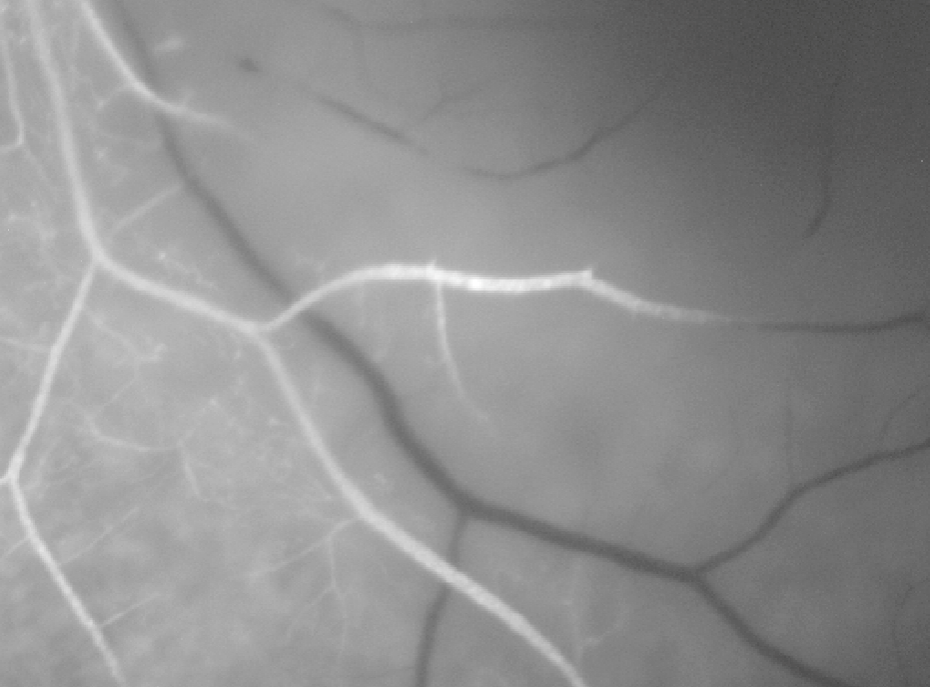
Magnification of fluorescein angiography where intraluminal microparticles can be evidenced

**Figure 5:**
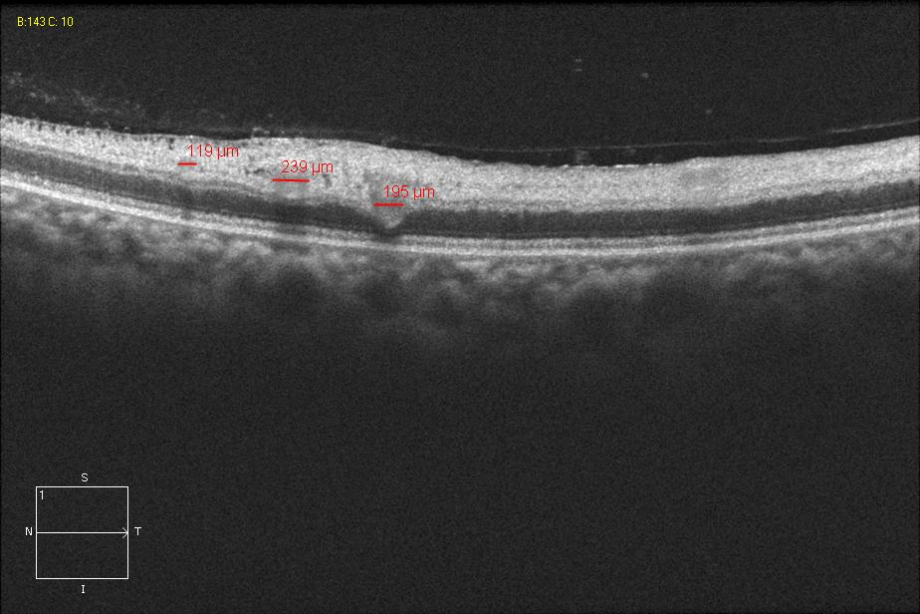
OCT image: Intraretinal hyporeflective spheres and their diameters.

**Figure 6:**
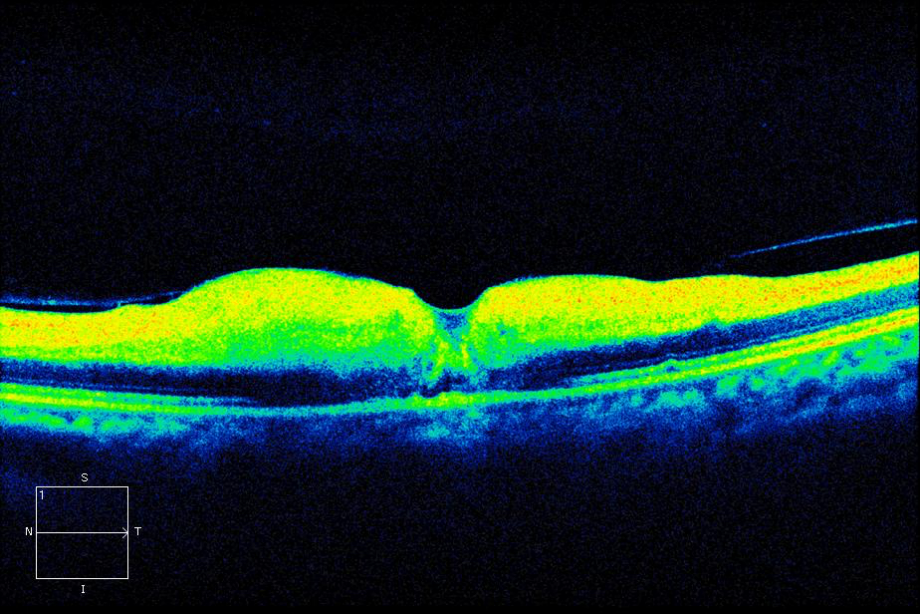
Macular OCT shows thickening of the internal retinal layers due to edema and ischemia

On the first postoperative day, the patient complained of poor vision in her left eye with no other associated symptoms. Brain MRI was performed to rule out intracerebral ischemia. Ophthalmology examination found no light perception, afferent pupillary defect, and a pale retina with arteriolar thinning and segmentation of macular vessels in the left eye (Figure 2 (Fig. 2)). Fluorescein angiography was performed and revealed an occlusion of the central retinal artery and the short posterior ciliary arteries. A severe interruption of blood flow was noticed on the arteriolar ramifications of the inferior retina (Figure 3 (Fig. 3)). Upon magnification, small hyperfluorescent, intraluminal spheres were noticed (Figure 4 (Fig. 4)). Optical coherence tomography (OCT) revealed hyporeflective, spherical images between 150–250 microns with posterior shadowing (Figure 5 (Fig. 5)) and thickening of the internal retinal layers due to edema and ischemia (Figure 6 (Fig. 6)). Multifocal ERG showed abnormal pattern secondary to an alteration affecting the ganglion cells of the papillomacular bundle. The patient was evaluated for a year without improvement of the visual acuity or neovascular complications in the anterior and posterior segment of the eye.

## Discussion

It is well known that paragangliomas may be hereditary and may be part of genetic syndromes. The sporadic form of carotid body paraganglioma is more common than the inherited variety and tends to occur slightly more often in women. Due to the proximity of the tumor to major vessels and nerves, the risk of associated morbidity and mortality is around 3–9% [9]. The risk seems to be significant when the tumor size is over 5 cm. Shamblin developed a classification system for the carotid body tumors [6]. CT is optimal for demonstrating the relationship of the tumor to adjacent structures and is useful in cases of suspected skull base invasion. Currently, digital subtraction angiography (DSA) is the gold standard that clearly delineates the details of the vascular structures adjacent to the tumor but also provides important information on collateral blood flow via the anterior, middle, and posterior cerebral artery from the contralateral side through the circle of Willis [10]. Carotid body tumors can be treated with surgical excision, radiation therapy, surgery and irradiation, or embolization and surgery, like our patient. Surgical removal is often associated with a significant intraoperative bleeding rate because of the vascular nature of the tumors. To reduce intraoperative blood loss, surgical time, and cranial nerve injury, preoperative de-vascularization with transarterial embolization and percutaneous embolization [11] has been proposed. This often remains incomplete because of the extensive vascular architecture and substantial arteriovenous shunting of these lesions. Successful embolization depends upon occlusion of all feeding vessels which, based upon prior studies, has shown to be high. Embolization is normally done using PVA with sizes ranging from 150 to 1000 microns. However, agents such as alcohol conjugates, liquid embolics (glue) and gel foam are also available. Complications of preoperative embolization vary. Minor complications include fever and facial pain, which are attributed to tumor ischemia and are usually transient. Major complications such as stroke may occur with the accidental introduction of emboli into the vertebrobasilar system via the ECA or its anastomoses with the ICA. Many possible dangerous anastomoses communicating the internal with the external circulation have been described [12]. Other documented risks include transient aphasia, carotid sinus syndrome, and catecholamine storm. To obtain the best results after supra-selective arterial embolization, some authors recommend surgical resection to be performed within the first 48 hours to minimize revascularization, edema, and recruitment of collateral tumor blood supply, prior to onset of significant local inflammatory reaction. There has been controversy concerning the usefulness of preoperative embolization. Some authors prefer routine preoperative embolization, particularly in larger tumors (Shamblin type II and III) [7] because it can lower blood flow, decrease tumor size, and facilitate tumor excision with less bleeding. Other schools disagree on preoperative routine embolization due to postembolization morbidity such as the potential risk of stroke by embolic particles. 

To our knowledge this is the first report of acute central retinal arterial occlusion secondary to paraganglioma embolization and excision with Embosphere^®^ and Contour^®^ microparticles. Due to the late onset of symptoms we believe this was a complication of the surgical procedure per se, in which, the surgical manipulation could have eased the liberation of particles to the circulation. It remains an interrogant as to why the particles embolized directly to the central retinal artery instead of other high-flow intracerebral branches. The patient’s OCT was conclusive on the presence of hyporeflective spherical particles that confirmed the nature of the embolization. Although a rare complication, we think it must be assessed with the patient as one of the possible complications prior to embolization and surgical resection procedures. 

## Conclusion

Central retinal artery occlusion should be assessed as a possible complication after embolization and surgical repair of head and neck paragangliomas.

## Notes

### Competing interests

The authors declare that they have no competing interests.
